# Collegial surface acting emotional labour, burnout and intention to leave in novice and pre‐retirement nurses in the United Kingdom: A cross‐sectional study

**DOI:** 10.1002/nop2.649

**Published:** 2020-10-15

**Authors:** Catherine Theodosius, Christina Koulouglioti, Paula Kersten, Claire Rosten

**Affiliations:** ^1^ School of Health Sciences University of Brighton Brighton UK; ^2^ Research and Innovation Department Western Sussex Hospitals NHS Foundation Trust Worthing UK; ^3^ NIHR Research Design Service South East & Centre for Health Research University of Brighton Brighton UK

**Keywords:** burnout, collegial emotional labour, cross‐sectional study, intention to leave, nurses, surface acting

## Abstract

**Aim:**

To investigate the relationship between surface and deep acting in nurses' patient‐focused and collegial emotional labour, with emotional exhaustion, depersonalization and personal accomplishment and intention to leave.

**Design:**

A cross‐sectional descriptive study using the Emotional Labour Scale, the Maslach Burnout Inventory and intention to leave Yes/No questions with 118 Registered Nurses to measure patient‐focused and collegial emotional labour, burnout and intention to leave.

**Results:**

Surface acting in patient‐focused *and* collegial emotional labour was found to have positive associations with burnout and intention to leave their current job. Only surface acting in patient‐focused emotional labour was positively associated with intention to leave the organization and/or the profession. The novice nurses carried out more deep acting collegial emotional labour than the pre‐retirement nurses.

**Conclusions:**

Collegial emotional labour is significant to nurses' intention to leave their current job but not their intention to leave the organization and/or the profession.

## 1 INTRODUCTION

Poor team‐working relationships have been identified as a contributory factor in nurse retention (Brewer et al., 2020; Hayward et al., 2016; Health Education England, 2014; Migration Advisory Committee, 2016). Bullying and workplace adversity is also a known factor underpinning poor retention of newly qualified nurses (Delasega, 2019; Laschinger et al., 2012; Laschinger & Grau, 2012). A key skill essential in promoting effective team working is collegial emotional labour (CEL)—the management of emotions to produce effective communication and team collaboration (Delgado et al., 2017; Theodosius, 2008, 2012). Current evidence identifies emotional labour as being positively correlated with burnout, and burnout out to intention to leave, amongst nurses (Na & Park, 2019). In the literature, there is no differentiation between emotional labour carried out for patient benefit (patient‐focused emotional labour (PF‐EL) and emotional labour carried out between colleagues (CEL). This paper discusses the quantitative findings of a cross‐sectional descriptive study investigating the relationship between surface and deep acting in PF‐EL and CEL, burnout and retention in newly qualified and pre‐retirement nurses in an acute care National Health Service (NHS) Trust in the South East of England.

## 2 BACKGROUND

### 2.1 Retention in nursing

Retention in nursing is not a new issue (Menzies, 1960). It is, however, critical as a lack of nurses is exacerbating retention problems at a time when demand for healthcare services has risen due to ageing populations with multiple and complex pathologies and large numbers of nurses reaching retirement age (Buchan et al., 2016; Kings Fund 2019). Indeed, the World Health Organization (2016) estimates that by 2030, the global shortfall of nurses and midwives will be around 7.6 million. In the United States, the Bureau of Labor Statistics (2018) estimates that currently around 1.13 million more nurses will be needed to avoid further shortages (US Bureau of Labor Statistics, 2018). In the UK, the Nursing Times reported that during “the first quarter of 2019–2020, 12% of full‐time equivalent registered nurse posts in the provider sector were empty, equating to 43,617 missing staff (Mitchell, 2019).” Indeed, the Kings Fund (Beech et al., 2019) latest report estimates that within 10 years there will be a shortfall of 108,000 fulltime equivalent nurses in the NHS. NSI Nursing Solutions, 2020 reporting on RN staffing in the United States found the turnover rate for bedside RNs grew to 17.2% equalling the highest rate in the past decade. In 2017, the Nursing and Midwifery Council (NMC) reported that the number of UK registrants leaving the profession had increased by 9% while the number of EU registrants leaving, exacerbated by Brexit, had increased by 67%. NHS Digital (2018) reported 1:10 nurses were leaving the profession, with over half of them under the age of 40. Nurse shortage, therefore, is not only about the baby boomer generation reaching retirement age.

### 2.2 Emotional labour, burnout and intention to leave

Emotional labour a term coined by Hochschild (1983) and applied to nursing in the UK by Smith (1992) and Theodosius (2008) link it to professional values and competencies and the giving of compassionate care. Emotional labour involves the ability to manage one's own emotions to facilitate a desired emotion response in others (Hochschild, 1983), such as feeling cared for in a patient, de‐escalating anger to resolve conflict or comforting someone in distress and is a key interpersonal skill for nurses. Feeling rules guide the nurse in knowing what emotions need managing and the degree of management required (Hochschild, 1983). Feeling rules such as being kind and compassionate, for example are stipulated in the Nursing and Midwifery Council Code (2018) in the UK. Emotional labourers deploy two main strategies: surface and deep acting. Surface acting is where the labourer displays feelings they know they do not have, suppressing their real emotions for the purposes of the other person (Hochschild, 1983). With deep acting, the labourer works on their emotions and induces within themselves the actual feelings required (Hochschild, 1983).

The relationship between emotional labour and burnout has previously been established (Brotheridge & Lee 2003, Erickson, 2009; Mann & Cowburn, 2005); specifically, studies report a positive correlation between surface acting emotional labour and burnout (Bartram et al., 2012; Brotheridge & Lee 2003; Grandey 2003). For example, Brotheridge and Grandey (2002:28) found that “surface acting correlated significantly with emotional exhaustion (*r* = 20; *p *< .01), depersonalization (*r* = 38; *p *< .01) and personal accomplishment (*r* = 18; *p* < .01) in the expected directions.” Bartram et al. (2012) did not differentiate between surface and deep acting strategies, but also found that emotional labour is positively associated with both burnout and intention to leave. Further, their study suggested that emotional labour significantly predicts intention to leave (*b* = 6.0, *t* = 4.5, *p* < .001) and burnout (*b* = 7.5, *t* = 13.2, *p* < .001). Karimi et al. (2014) found that the more emotional labour dissonance experienced by nurses, the lower the level of well‐being (*b* = 0.18; *p* < .01) and a higher level of job stress (*b* = 0.35; *p* < .01). Schmidt and Diestel 's (2014) study showed that surface acting took more cognitive control than deep acting and was related to burnout, depression and absenteeism more than deep acting. Pisaniello (:589) compared emotional labour (as a work requirement), with emotional work, (as privately given) and found that “suppressing negative emotion is a risk factor for stress, personal burnout and job dissatisfaction, whereas the use of emotion regulation strategies (i.e. surface acting) in addition to emotion job requirements is linked to increased stress and work and patient‐related burnout.” Others have established a predictive relationship between surface acting and intention to leave (Chau et al., 2009), between emotional labour, job satisfaction and staff well‐being in nursing (Chou et al., 2012; Karimi et al., 2014). These are significant findings as rates of burnout are high. For example, in a European wide survey, where on average 28% of nurses reported burnout, in the UK it was as high as 42% (Health Education England, 2014).

### 2.3 Collegial relationships and burnout

The positive correlation between surface acting and nurse burnout with intention to leave and staff turnover is a consistently reported international phenomena (Chou et al., 2012; Erickson, 2009; Karimi et al., 2014; Mann & Cowburn, 2005; Schmidt & Diestel, 2014; Yoon & Kim, 2013). In fact, it is not particular to nursing with the same phenomena reported in teaching, law, the police, tourism and hospitality (Anleu & Mack, 2005; Chau et al., 2009; Lee, 2019; Schaible & Gecas, 2010). This suggests that it is a difficulty confronting nursing as a profession rather than being specific to systems of healthcare delivery or social cultures. Indeed, Maslach and Leiter (2016:103) note that burnout is “an occupational hazard for various people‐oriented professions, such as human services, education and health care” because “therapeutic or service relationships that such providers develop with recipients require an ongoing and intense level of personal, emotional contact.” This orientation towards caring relationships is particularly relevant in nursing due to high levels of interaction between nurses and patients and high levels of public and professional expectation that these are positive (Delgado et al., 2017; Jeung et al., 2018). There is an assumption, therefore, that it is emotional labour carried out within the caring relationship that is linked to burnout—that is patient‐focused emotional labour.

The intention to leave and turnover literature gives less attention to emotional labour. Rather, the focus is on leadership, team working, patient acuity and lack of resources. Hayward et al. (2016), for example identified professional relationships as being key to nurses' decisions to leave, findings supported by Viotti et al. (2018) and Heinen et al. (2013) studies, which found poor team working, bullying and incivility between nurses were a predictor of intention to leave. Indeed, studies exploring the role of emotional labour in leadership, teams and organizations found that emotional labour between colleagues is linked to intention to leave (Becker et al., 2018; Halter et al., 2017; Heinen et al., 2013; Iszatt‐White, 2013; Viotti et al., 2018).

There is particular concern that stress, burnout and turnover rates are high in novice nurses in their first and second year post qualification (Health Education England, 2014). The transition period during early practice experiences has been identified as exposing them to poor working relationships, poor leadership and workplace bullying (Jackson et al., 2011; Kings Fund 2019; Thomas et al 2015). Such exposure is damaging to their developing professional resilience and may contribute towards increased turnover rates and intention to leave. Exploring the significance of CEL at the outset of a professional career is valid, therefore, given the high levels of attrition, stress and role adjustment during this time (Caliskan & Ergun, 2012; Duchscher, 2009; Ferguson, 2011).

Middleton (2017), however, asks why the nursing profession is not doing more to prevent experienced nurses taking early retirement. While most retire for financial reasons, those who choose to remain do so because of the satisfaction gained from patient interaction and patient care (Uthaman et al.'s, 2016). However, this was found to be dependent on good working relationships and leadership (ibid). Indeed, Daouk‐Öyry et al. (2014) in their systematic review found that management style and interpersonal relationships between colleagues were significant variables to turnover.

It is possible, therefore, that surface acting in CEL may be positively associated with burnout and intention to leave. It is also possible that there may be a difference between novice and pre‐retirement nurses.

### 2.4 Research questions

1. In novice and pre‐retirement nursing staff
  - Is there a relationship between *patient‐focused emotional labour* and burnout (inclusive of emotional exhaustion, depersonalization and reduced personal accomplishment) and their intention to leave their job or/and the nursing profession?
  - Is there a relationship between *collegial emotional labour* and burnout and their intention to leave their job or/and the nursing profession?
  - Are there any differences between patient‐focused and collegial emotional labour in the relationship (if found) with burnout and their intention to leave their job or/and the nursing profession?
2. Are there any differences between novice and pre‐retirement staff and patient‐focused emotional labour, collegial emotional labour, burnout and intention to leave?

## 3 METHODS

### 3.1 Design

The study employed a cross‐sectional descriptive design using three self‐report measures: the Emotional Labour Scale (ELS) (Brotheridge and Lee 2003), the Maslach Burnout Inventory (MBI) (Maslach & Jackson, 1986; Maslach et al., 1996) and a 3‐item Yes/No Turnover measure (Griffeth et al., 2000). This design was chosen because the literature suggests there is a positive correlation between surface acting and emotional exhaustion and depersonalization (Brotheridge & Lee 2003; Erickson & Grove, 2002; Grandey, 2002; Schmidt & Diestel, 2014), key factors predicating burnout.

### 3.2 Participants

In this study, novice nurses were defined as those in receipt of their Nursing and Midwifery Council PIN [denoting they can practice as Registered Nurses] for a minimum of 6 months and employed by the NHS Trust for a maximum of 2 years and had completed the preceptorship programme supporting transition run by the Trust for newly qualified nurses. Pre‐retirement nurses were defined as those who had been registered and practicing for a minimum of 5 years and were 50 years old or over. Two groups, novice nurses and pre‐retirement nurses, were recruited from three acute care hospitals in a NHS Trust in the South East of England between November 2017–April 2018. Nurses were included if they met the definition for a novice or pre‐retirement nurse. There were no exclusion criteria.

During this 6‐month period, the Trust employed 1,971 nurses. They had a reported staff turnover of 8.44% and 656 staff were eligible to retire, figures matching the national picture (NHS Improvement 2018). Using the sample size calculator http://www.sample‐size.net/correlation‐sample‐size/ and the correlation coefficient of 0.38 (Brotheridge & Lee 2003) between the Emotional Labour Scale (ELS) and the MBI, for 95% confidence intervals, the level of significance was set at *α* = 0.05, power *β* = 80%. Fifty‐two nurses per group were required for hypothesis testing, a minimum sample total of 104 (*N* = 104).

A total of 118 nurses were recruited across sites, 58 novice nurses (*N* = 58) and 60 pre‐retirement nurses (n‐60). All nurses who met the inclusion criteria were contacted via email and sent the participant information sheet, consent forms and the questionnaire. In addition, hard copies of these documents were distributed to all practice areas and collected one week later. To encourage participation in the questionnaire, a £5 honorarium was given to all nurses who completed the questionnaire.

### 3.3 The measures

The nurses reported demographic information about their age, gender, employment status, banding and years of experience since receipt of their PIN. The ELS is a validated 15‐item self‐report questionnaire with 6 subscales (frequency, intensity, variety and duration of emotions and surface and deep acting). It is designed to understand the type (surface or deep acting) and degree of emotional labour the participant is carrying out while interacting with patients and colleagues during their everyday work at the hospital. Example item: How often do you “Resist expressing your true feelings?” It employs a 5‐point Likert scale ranging from 1 (never) to 5 (always) (scoring ranging between 15–75). A total score is calculated by summarizing all the subscales inclusive of surface and deep acting. It has good internal consistency (Cronbach's *α* 0.74–0.91) and construct validity (Brotheridge and Lee 2003).

The Maslach Burnout Inventory (MBI) is a validated measure of burnout that occurs due to occupational stress (Maslach & Jackson, 1986; Maslach et al., 1996). It has 22 items divided into three subscales measuring three key aspects of burnout: emotional exhaustion (nine items), depersonalization (five items) and reduced personal accomplishment (eight items) (Maslach et al., 1996). The MBI has good internal reliability (Cronbach's *α* 0.71–0.90) (Maslach et al., 1996:198). The reliability coefficients for the subscales were 0.90 for emotional exhaustion, 0.79 for depersonalization and 0.71 for personal accomplishment (Maslach et al., 1996:198). The items are rated using a Likert scale ranging from 0, (never)—6 (everyday). Example items: Please rate how frequently you feel: “I feel emotionally drained from my work” or “I can easily create a relaxed atmosphere with my recipients.” Convergent and discriminant validity of ELS with the MBI showed that both emotional exhaustion and depersonalization from the MBI were significantly positively correlated with the surface acting subscale from the ELS (*R* = 0.38) (Brotheridge & Lee, 2003: 372).

The literature on turnover in nursing suggests that thinking about leaving—intention to leave—is the most significant predictor of actual quitting (Mobley et al., 1978). Yes = 1/No = 0 questions stating intention to quit: (a) their current job, (b) the organization and (c) the nursing profession, were used to measure this. The specific questions asked were: “Have you thought about leaving your current job?” “Have you thought about leaving the organization?” and “Have you thought about leaving the Nursing Profession?”

### 3.4 Ethics

The University and the Health Research Authority scrutinized the study and granted ethical permissions for it to go ahead (IRAS Project ID 223834). No patients were included in this study.

### 3.5 Analysis

The statistical software SPSS version 22 was used for the analysis. Frequency distributions and descriptive statistics (means, medians, modes and standard deviations) were calculated on all demographic and study measures. Data were analysed for variance and distribution anomalies (e.g. skewness, outliers) that could distort the analyses. Such distortions were not found, so variable transformation was not used, also there were no missing data that had to be handled in the data set. Data were further analysed using the appropriate parametric and non‐parametric tests. Relationships between continuous variables were explored using Pearson's correlation coefficients (*r*) and differences between groups with independent samples *t* tests or chi‐squared tests. As described in the research questions, differences between novice and experienced nurses were explored in relation to the three main outcomes: emotional labour, burnout and intention to leave work. The data were analysed by the team and independently verified by an expert statistician.

## 4 RESULTS

### 4.1 Participant characteristics

Across the total sample (*N* = 118), there were eight men and 109 women; seventy‐nine worked fulltime and thirty‐six worked part‐time. In the NHS, novice nurses are appointed entry‐level jobs at band 5 (*N* = 70) progressing to band 6 (*N* = 23) after a few years. Band 7 nurses (*N* = 18) usually hold managerial roles equivalent to ward managers clinical nurse specialists. Band 8 nurses (*N* = 7) are senior managers in the NHS. Participants' age ranged between 21–50 years (*N* = 66) and 51–70 years of age (*N* = 51). There were 58 in the novice group (*N* = 58) and 60 in the pre‐retirement group (*N* = 60). All novice nurses had less than 2 years of experience (as per eligibility criteria for the study), and most experienced nurses (*N* = 50) had => 20 years of working experience.

### 4.2 Questions 1.i and ii

A significant positive correlation was found between PF‐EL and both emotional exhaustion (*r* = 0.361, *N* = 118, *p* < .001) and depersonalization (*r* = 0.359, *N* = 117, *p* < .001). A significant negative correlation was found between PF‐EL and personal accomplishment (*r* = −0.350, *N* = 117, *p* < 0). A significant positive correlation was found between CEL and both emotional exhaustion (*r* = 0.363, *N* = 118, *p* < .001) and depersonalization (*r* = 0.355, *N* = 117, *p* < .001). A significant negative correlation was found between CEL and personal accomplishment (*r* = −0.251, *N* = 117, *p* < .01). Table 1 presents the means and standards deviations of emotional labour and burnout for both the novice and pre‐retirement nurses.

**TABLE 1:** Mean and standard deviations between novice and pre‐retirement nurses

	Group	Mean	Standard deviation
PFEL‐SA	Novice	9.69	2.44
	Pre‐Retirement	9.07	2.52
PFEL‐DA	Novice	9.78	2.55
	Pre‐Retirement	8.98	2.99
CEL‐SA	Novice	9.28	2.38
	Pre‐Retirement	8.75	2.32
CEL‐DA	Novice	9.90	2.83
	Pre‐Retirement	8.65	3.06
Emotional exhaustion	Novice	24.45	12.28
	Pre‐Retirement	23.15	12.74
Personal accomplishment	Novice	34.78	8.20
	Pre‐Retirement	37.56	6.82
Depersonalization	Novice	5.97	5.19
	Pre‐Retirement	5.07	4.88

Abbreviations: CEL‐DA, collegial emotional labour‐deep acting; CEL‐SA, collegial emotional labour‐surface acting; PFEL‐DA, patient‐focused emotional labour‐deep acting; PFEL‐SA, patient‐focused emotional labour‐surface acting.

Only two statistically significant differences were found. The novice nurses reported a higher mean score than the pre‐retirement nurses on deep acting CEL [*t*(116) = 2.293, *p* < .05]. There was also a significant difference between the scores of the two groups on personal accomplishment [*t*(115) = −1.997, *p* < .05], with pre‐retirement nurses having a higher mean score than novice nurses.

### 4.3 Question 1.iii

More pre‐retirement nurses reported thinking about *leaving their organization* than novice nurses [*Χ* ^2^ (1, *N* = 118) = 4.861, *p* < .05], and more pre‐retirement nurses thought about *leaving the profession* than novice nurses [*Χ* ^2^ (1, *N* = 118) = 8.102, *p* < .01]. No significant relationship between intention to leave and *their current job* was found in the pre‐retirement group and novice nurses [*Χ* ^2^ (1, *N* = 118) = 0.521, *p* > .05].

Table 2 presents the statistically significant differences between the scores of the two “intention to leave *the job*” groups (group Yes and group No) with PF‐EL and surface acting. Those intending to leave their job scored higher in PF‐EL and surface acting than those intending to stay [*t*(116) = −3.649, *p* < .001]. In addition, those intending to leave their job scored higher in CEL and surface acting than those intending to stay [*t*(116) = −2.443, *p* < .05]. There were also significant differences between the groups with emotional exhaustion, [*t*(116) = −6.062, *p* < .001] and those intending to leave their job scoring higher than those intending to stay and depersonalization [*t*(115) = −2.858, *p* < .001] and those intending to leave their job scored higher than those intending to stay. In personal accomplishment [*t*(115) = 2.034, *p* < .001], those intending to leave their job scored lower than those intending to stay (See Table 3).

**TABLE 2:** Novice and pre‐retirement nurse's intention to leave

Intention to leave	Group	Yes		No	
		Frequency	Per cent	Frequency	Per cent
Job	Novice	30	25.4	28	23.7
	Pre‐Retirement	35	29.7	25	21.2
Organization	Novice	24	20.3	34	28.8
	Pre‐Retirement	37	31.4	23	19.5
Profession	Novice	16	13.6	42	35.6
	Pre‐Retirement	32	27.1	28	23.7

**TABLE 3:** Intention to Leave and surface and deep acting in patient‐focused and collegial emotional labour

	Intention to leave current job	Number	Mean (Standard Deviation)	Intention to leave Organization	Number	Mean (Standard deviation)	Intention to leave profession	Number	Mean & Standard deviation
PFEL‐SA	Yes	65	10.09 (2.26)	Yes	61	9.90 (2.49)	Yes	48	9.71 (2.59)
	No	53	8.49 (2.56)	No	57	8.81 (2.39)	No	70	9.13 (2.42)
PFEL‐DA	Yes	65	9.37 (2.42)	Yes	61	9.33 (2.60)	Yes	48	8.94 (2.58)
	No	53	9.38 (3.22)	No	57	9.42 (3.02)	No	70	9.67 (2.92)
CEL‐SA	Yes	65	9.48 (2.06)	Yes	61	9.30 (2.22)	Yes	48	9.00 (2.37)
	No	53	8.43 (2.58)	No	57	8.70 (2.47)	No	70	9.01 (2.36)
CEL‐DA	Yes	65	9.32 (2.89)	Yes	61	9.23 (2.93)	Yes	48	8.73 (3.05)
	No	53	9.19 (3.16)	No	57	9.30 (3.11)	No	70	9.63 (2.94)
Emotional exhaustion	Yes	65	29.29 (11.80)	Yes	61	28.30 (12.09)	Yes	48	28.69 (11.37)
	No	53	17.04 (9.74)	No	57	18.96 (11.09)	No	70	20.43 (12.16)
Personal accomplishment	Yes	65	34.89 (8.04)	Yes	61	34.58 (8.48)	Yes	48	34.60 (8.16)
	No	53	37.74 (6.86)	No	57	37.86 (6.27)	No	70	37.28 (7.10)
Depersonalization	Yes	65	6.69 (5.19)	Yes	61	6.63 (5.33)	Yes	48	6.69 (5.55)
	No	53	4.09 (4.49)	No	57	4.33 (4.45)	No	70	4.70 (4.51)

Abbreviations: CEL‐DA, collegial emotional labour‐deep acting; CEL‐SA, collegial emotional labour‐surface acting; PFEL‐DA, patient‐focused emotional labour‐deep acting; PFEL‐SA, patient‐focused emotional labour‐Surface acting.

Furthermore, significant differences between the scores of the two “intention to leave *the organization*” groups can be seen with PF‐EL and surface acting, *t*(116) = −2.432, *p* < .05, with those intending to leave the organization scoring higher than those intending to stay. There were no significant differences between CEL, surface acting and intention to leave the organization. However, those intending to leave the organization scored higher than those intending to stay on emotional exhaustion, *t*(116) = −4.359, *p* < .001 and on depersonalization, [*t*(116) = 2.366, *p* < .05]. Those intending to leave the organization scored lower on personal accomplishment than those intending to stay [*t*(116) = −2.527, *p* < .05].

There were also significant differences between the scores of the “intention to leave *the profession*” on emotional exhaustion, [*t*(115) = −3.72, *p* < .001], with those intending to leave the profession scoring higher than those intending to stay; and on depersonalization, [*t*(115) = −2.137, *p* < .05], with those intending to leave the profession scoring higher than those intending to stay.

## 5 DISCUSSION

Our results reaffirm the positive association between surface acting and burnout, similarly to Brotheridge and Grandey (2002) in service work, Zhang and Zhu (2008) in teachers and Cheng et al. (2013) and Chou et al. (2012) in nurses, who found a significant association between surface acting/faking emotions and burnout. While we found no statistical significance *between* PF‐EL and CEL, there was a positive association between surface acting in both PF‐EL *and* CEL and intention to leave their current job. Chou et al. (2012), Grandey's (2003) and Zhang & Zhu (2008) and studies, respectively, found that surface acting emotional labour is positively related to emotional exhaustion and negatively related to job satisfaction. Bartram et al. (2012) and Cheng et al. (2013) found that in nursing, surface acting and burnout predicted intention to leave. Further, direct patient care and positive professional relationships between colleagues were found by Hayes et al (2010) to be significant factors in job satisfaction. This study's findings about CEL and PF‐EL as being positively associated with burnout and intention to their current job therefore contributes to these findings by identifying the significance of both CEL and PF‐EL.

There was no statistically significant difference between CEL and intention to leave the organization or between CEL and intention to leave the profession. There was a significant association between PF‐EL surface acting and intention to leave the organization, but not between PF‐EL surface acting and intention to leave the profession. Leaving the profession altogether was only of significance with the pre‐retirement nurses and was positively associated with burnout factors only. The difference on intention to leave between the two groups could also be explained from the fact that 50 of the nurses in the experienced group had more than 20 years of working experience and were possibly eligible for retirement based on their NHS employment contract. Leiter and Maslach (2009) found in their study that younger more novice nurses were more vulnerable to job burnout than their senior counterparts. Our study, however, has found that *both* novice and experienced nurses were likely to report burnout due to CEL *and* PF‐EL. Leiter et al, however, also reported that younger nurses found their work less satisfying in comparison with more experienced nurses. This may reflect our finding that pre‐retirement nurses experience more personal accomplishment than their novice counterparts.

These differences potentially suggest that when nurses consider leaving their current job, both PF‐EL and CEL surface acting contribute to that decision, whereas when considering leaving the organization, only PF‐EL surface acting is significant. The positive association between surface acting in CEL and intention to leave their current job may reflect Hayward et al.'s (2016) finding that poor working relationships with colleagues are key to when nurses make the decision to leave. Equally, the positive association between surface acting and PF‐EL may reflect findings on the importance of good nurse–patient interaction and good patient outcome with satisfaction at work (Chou et al., 2012; Karimi et al., 2014; Lu et al., 2019; Pisaniello, 2012. When this is impaired, such as when nurses use surface acting, it may influence their intention to leave their job. Interestingly Yang and Chang (2008:885) found “a positive relationship between job satisfaction and organizational commitment but that surface acting has a negative influence on organizational commitment, indicating that when nurses perform emotional labour that differs from their inner feelings, rather than affect their degree of job satisfaction it depresses their organizational commitment.” Their findings are supported Walsh and Bartikowski (2013) who found that only surface acting had a negative impact on nurse job satisfaction and increased their intention to leave their job and Gountas et al. (2014) who found that job satisfaction acted as a mediator between surface acting and intention to leave.

In this study, we set out to identify whether there was a difference between two types of emotional labour. What distinguishes PF‐EL from CEL are the feeling rules which shape the purpose, content and outcome of the emotional labour. For example, PF‐EL is concerned with the therapeutic relationship, based on feeling rules and social values concerned with compassion and kindness and the understanding that nurses can help alleviate suffering through their acts of emotional labour (Smith 1992; Theodosius, 2008). In CEL, the feeling rules are instrumental to facilitating team working, displaying status and ensuring that everyday practical patient care differing practitioners set out to accomplish are achieved efficiently (Theodosius, 2008). Because feeling rules are localized in CEL, they are vulnerable to being shaped by the social environment and can be based around negative communication patterns, bullying and harassment and the assertion of status (Delgado et al., 2017; Theodosius, 2008, 2012). Such differences between types of emotional labour are to do with its meaning and social purpose—*the why and* the social context where the emotional labour takes place. Interestingly, in this study, CEL was found to have a significant positive association to intention to leave the current job, possibly indicating the impact of localized practice environments, but had no association when concerned with either the organization and/or the profession which represent larger social contexts and structures. Further, novice nurses were carrying out significantly more deep acting CEL than their pre‐retirement counterparts. This may reflect the importance of displaying status in CEL as junior nurses are expected to do with their senior colleagues (Theodosius 2008).

### 5.1 Significance

The original contribution of this study is that surface acting CEL in nursing is associated with burnout and nurse intention to leave their current job in addition to surface acting PF‐EL. That CEL surface acting is linked to intention to leave their current job but not to nurses' intention to leave the organization or the profession suggests that its main significance is to high levels of nurses' job turnover rates. This is a significant contribution to the literature, especially as CEL is associated with burnout in both novice *and* pre‐retirement nurses. This suggests that when considering retention strategies organizations need to consider the impact of CEL and PF‐EL. This might be of particular relevance in pre‐retirement nurses who choose to take early retirement from their current job and then work part‐time as a band 5 on the bank. Further, this might also be of particular relevance in novice nurse turnover rates especially as they appear to be carrying out significantly more CEL than the pre‐retirement nurses. Indeed, studies by Lavoie‐Tremblay et al. (2011) and Takase et al (2009) found that the need for professional recognition was a significant factor in turnover intention in younger nurses.

More pre‐retirement nurses than novice nurses had thought about leaving the organization and the profession, this is to be expected as by definition, they are approaching retirement age. However, it is interesting to note that burnout factors of emotional exhaustion and depersonalization were significant to those considering leaving their current job and those leaving the organization or profession.

These findings support Uthaman et al.'s (2016) who found that pre‐retirement nurses who choose to remain do so because of the satisfaction gained from patient interaction and patient care, but that this was dependent on good working relationships and leadership. This suggests that addressing burnout, particularly emotional exhaustion and depersonalization, is of significance to discourage pre‐retirement nurses to retire early. Further, understanding the role of CEL in pre‐retirement nurses intending to leave their current job may also be a contributing factor. Future studies with the inclusion of larger samples would help to further understand these complex relationships by conducting multivariate statistical analysis and exploring specific mediating and moderating relationships.

### 5.2 Limitations

This is a local study which had an impact on our ability to generalize. Due to the cross‐sectional nature of the study, it is not possible to make statements about cause and effect between surface acting, burnout and intention to leave. There was an excellent uptake, so achieving the correlation coefficient of 0.38, between the ELS and MBI for 95% confidence intervals (Brotheridge & Lee 2003) requiring 104 participants, was exceeded (*N* = 118). Further, our results are in a similar vein to other studies investigating the relationship between surface acting emotional labour and burnout as outlined above in the discussion (Bartram et al., 2012; Brotheridge & Grandey, 2002; Cheng et al., 2013). A multi‐centre longitudinal study would be needed to test the significance of surface acting in CEL and intention to leave their current job.

## 6 CONCLUSION

Surface acting collegial emotional labour is significant to nurses' intention to leave their current job but not their intention to leave the organization and/or the profession. This is the case for both novice nurses and pre‐retirement nurses. Surface acting patient‐focused emotional labour on the other hand is positively associated with intention to leave the organization and/or the profession. This suggests that collegial emotional labour is significant to nurses' job turnover, but that patient‐focused emotional labour is significant to nurse retention.

## CONFLICT OF INTERESTS

There is no conflict of interests to declare for the named authors of this article.

## Data Availability

The data supporting the findings of this study are not publicly available due to privacy or ethical restrictions. Enquiries about the data can be made to the corresponding author Dr Catherine Theodosius.
